# Playing “hide and seek” with the Mediterranean monk seal: a citizen science dataset reveals its distribution from molecular traces (eDNA)

**DOI:** 10.1038/s41598-023-27835-6

**Published:** 2023-02-14

**Authors:** Elena Valsecchi, Giacomo Tavecchia, Ginevra Boldrocchi, Emanuele Coppola, Denise Ramella, Livia Conte, Monica Blasi, Antonia Bruno, Paolo Galli

**Affiliations:** 1 Department of Environmental and Earth Sciences, University of Milano-Bicocca, Piazza della Scienza 1, 20126 Milan, Italy; 2 MaRHE Center, Magoodhoo Island, Faafu Atoll Republic of Maldives; 3 Mediterranean Institute for Advanced Studies (IMEDEA-CSIC/UIB), IMEDEA-C/Miquel Marquès, 21, 07190 Esporles, Balearics Islands Spain; 4 Department of Human Sciences, Innovation and Territory, University of Insubria, Via Valleggio 11, Como, Italy; 5 One Ocean Foundation, Via Gesù 10, Milan, Italy; 6 Gruppo Foca Monaca APS, Via Carlo Emery 47, 00188 Rome, Italy; 7 Filicudi Wildlife Conservation, Stimpagnato, 98055 Filicudi, Lipari (ME) Italy; 8 Department of Biotechnology and Biosciences, University of Milano-Bicocca, Piazza della Scienza 2, 20126 Milan, Italy

**Keywords:** Ecology, Zoology, Ecology, Ocean sciences

## Abstract

Animal conservation relies on assessing the distribution and habitat use of species, but for endangered/elusive animals this can prove difficult. The Monk Seal, *Monachus monachus*, is one of the world's most endangered species of pinniped, and the only one endemic to the Mediterranean Sea. During recent decades, direct observations have been few and scattered, making it difficult to determine its distribution away from the Aegean Sea (core distribution area of the post-decline relict population). This study relies on environmental DNA (eDNA) analysis to detect the presence of the Monk Seal in 135 samples collected in 120 locations of the central/western Mediterranean Sea, spanning about 1500 km longitudinally and 1000 km latitudinally. A recently described species-specific qPCR assay was used on marine-water samples, mostly collected during 2021 by a Citizen Science (CS) project. Positive detections occurred throughout the longitudinal range, including the westernmost surveyed area (Balearic archipelago). The distribution of the positive detections indicated six “hotspots”, mostly overlapping with historical Monk Seal sites, suggesting that habitat-specific characteristics play a fundamental role. We applied single-season occupancy models to correct for detection probability and to assess the importance of site-specific characteristics. The distance from small islets and protected (or access-restricted) areas was correlated negatively with the detection probability. This novel molecular approach, applied here for the first time in an extensive CS study, proved its potential as a tool for monitoring the distribution of this endangered/elusive species.

## Introduction

Conservation biology hinges upon understanding the distribution and abundance of threatened species in order to draw appropriate conservation measures. However, assessing the distribution of elusive and endangered species is still challenging due to the difficulty of conducting direct observations, which are not numerous and are scattered over a large time frame, making classical survey methods of little use^1^ and resulting in a major lack of data^2^. This is particularly evident for the Mediterranean Sea, which is widely recognized as an important hotspot for marine biodiversity^3^, yet whose critical habitats for endangered species still need to be identified for conservation purposes^4–7^.

One of the most elusive marine mammals in the Mediterranean Sea is the Monk Seal, *Monachus monachus*, which is the only pinniped endemic to the Mediterranean Sea^8^. This species is listed as Endangered by the IUCN Red List and considered one of the most endangered pinniped species in the world^8^. Once abundant in the basin, the Monk Seal was eradicated from most of its former range well before the Second World War^9,10^. This species was exploited for subsistence needs, commercial harvest for skin and oil, and persecuted as a competitor for fishery resources and because it caused actual and perceived damage to fishing gear^10–12^. In the twentieth century, the decline of Monk Seals has been linked to habitat deterioration, destruction, and fragmentation, as well as to deliberate aggression by fishermen resulting in animal mortality, and to bycatch^8^. Despite verging on the brink of extinction, conservation measures introduced for its protection over the past 30 years have led to a small increase in its known subpopulations^8^. However, apart from its core distribution area in the Aegean Sea, direct observations are still relatively few, and thus the Monk Seal’s distribution, home range, habitat use, and seasonal movements remain poorly known^13^, even if a recent increase in sightings seems to suggest a slow but gradual recovery in population numbers^14,15^.

In this context of paucity of data, genetic analyses of wild animals’ traces such as scat, hair or fecal samples have provided a valuable non-intrusive method to study the presence and density of elusive species^16,17^. Nowadays, methodological enhancements in the field of molecular ecology allow us to detect genetic material (environmental DNA, eDNA) shed into the water by any marine organism, overcoming challenges of traditional monitoring. Despite some intrinsic limitations, such as the difficulty in determining the geographic origin and number of individuals releasing DNA traces, whose fate due to both degradation and transport is still not fully understood, the utilization of eDNA techniques has the potential to assess marine biodiversity, including rare, cryptic, or vulnerable marine mammal species. This approach may both complement existing surveys and map the geographic distribution of species over long time periods and across large spatial scales^18–20^.

Moreover, the easy sampling process makes eDNA analyses suitable for citizen-science (CS) projects—i.e. the active participation and engagement of the general public in scientific research—which has increasingly proven to be a rich and valuable source of information in defining species’ distribution^7,21–23^. In this context, environmental DNA coupled with citizen science in the Mediterranean Sea have the potential to enable wide geographical areas to be sampled simultaneously^24,25^, as this basin is one of the most densely inhabited in the world and its coasts are hotspots for tourism, representing a perfect case scenario for obtaining new information for this cryptic pinniped.

Although the fate and persistence of eDNA in the aquatic environment is still not fully understood, since it depends also on the source organism, as well as being affected by many biotic (e.g. entering the food web) and abiotic (e.g. UV, currents) factors^26–31^, it can still provide presence-absence data witnessing the passage of elusive species, such as the Monk Seal^32^.

Therefore, the aim of the present study is to detect molecular traces (eDNA) of the Monk Seal in the central and western Mediterranean Sea and to infer insight on habitat use and population abundance from samples of marine water collected by research institutions, as well as non-profit organizations and volunteers within the framework of the *Spot the Monk* 2021 initiative (Supplementary Appendix 1S). Moreover, the distribution of absence/presence data of molecular signals was also used to define its range boundaries, elucidate occurrence and detection estimates, identify ecological (e.g. foraging) corridors and pinpoint hotspot areas deserving future systematic monitoring.

## Results

### How widespread is the Monk Seal population in the central Mediterranean?

**Figure 1:**
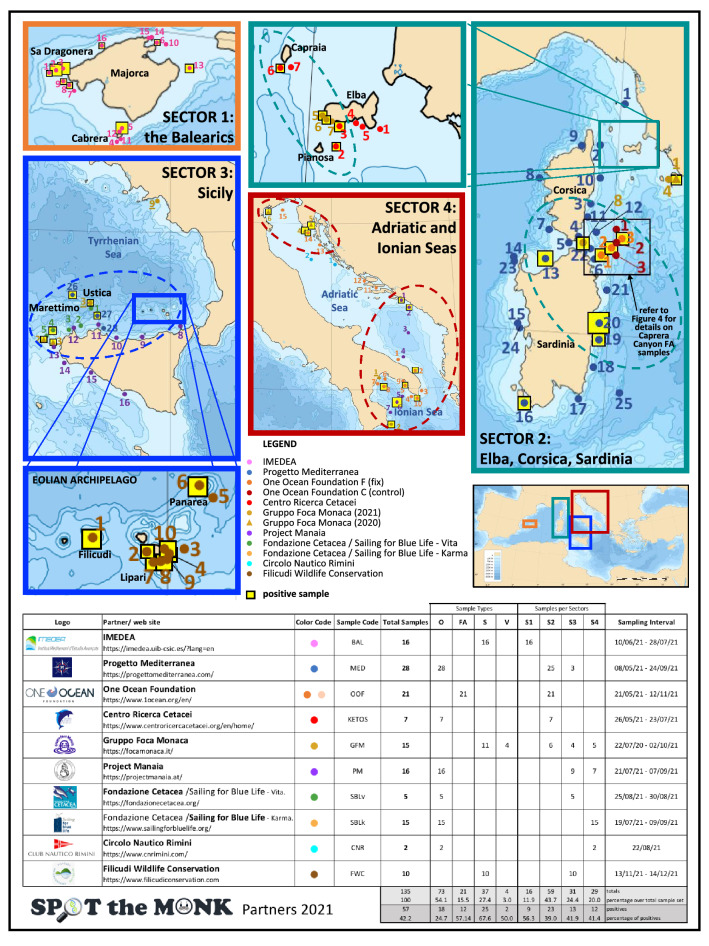
Maps of the four surveyed sectors showing the 120 points where samples (n=135) were collected. Sectors’ frames are colour-coded and their geographical position in relation to the Mediterranean Sea is shown in the map at the bottom right. For Sectors 2 and 3 a sub-map (with the same colour-code as the sector) is given to illustrate the samples taken from restricted areas. Sampling points surrounded by a yellow square indicate samples that tested positive for the presence of Monk Seal DNA in at least one of the experimental replicates: the size of the yellow square is proportional to the number of positive responses(the bigger it is the stronger the signal). The dashed lines delimit areas (hotspots) where multiple positive samples were observed and which would deserve continuous monitoring. The four underscored sample-numbers indicate V samples (validation samples) collected after a reported sighting, under the GFM coordination. The bottom table lists all the Spot the Monk partners contributing to the collection of samples, and their sampling effort (detailed per sample category, sector and sampling period). The Mediterranean Sea Bathymetry map was retrieved, and subsequently cropped, from https://commons.wikimedia.org.

**Figure 2:**
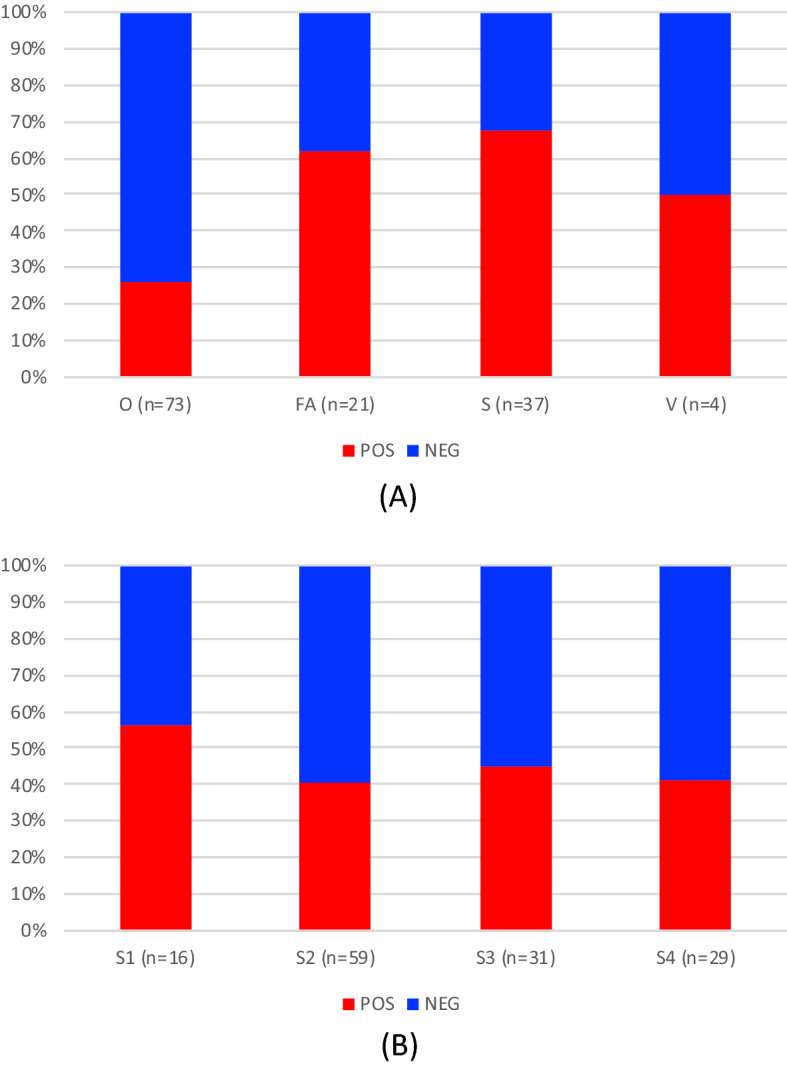
Incidence of positive responses (Monk Seal DNA detection in at least one sample replicate) across sample types (**A**) and geographic sectors (**B**). In the top graph, O = Opportunistic, FA = Focus Area, S = Surveillance , V = Validation samples. In the bottom graph S1 = Balearic Islands; S2 = Corsica, Sardinia and Tuscany archipelago, S3 = Sicily, S4 = Ionian and Adriatic seas.

The number of samples positive to Monk Seal DNA were 57 (42.2%,) out of 135 samples analysed (Supplementary Table 1S). Positive samples were found in all four sample categories and four geographic sectors (Figs. 1 and 2). The sample classes with the highest and lowest incidence of positive samples were the ‘Surveillance’ (67.57%, n = 25 out of 37) and ‘Opportunistic’ (24.66%, n = 18 out of 73) classes (Fig. 2A) respectively. The remaining two sample classes produced a proportion of positive results of 50% or more (‘Focus-Area’ and ‘Validation’ samples: 57.14% and 50%, respectively). We detected six “hotspots”, i.e. areas where multiple positive samples clustered (Fig. 1): one includes the whole Balearic sector (S1); one in the Tuscan archipelago and the western side of Elba island (S2); one in north-eastern/eastern Sardinia (S2); one embracing the complex system of archipelagos that lap the northern and western coast of Sicily (S3); one in the western Ionian and lower Adriatic seas (S4) and the last one in the upper Adriatic sea, around the Istrian peninsula (S4).

As expected, when the detection probability is accounted for, the model-averaged estimate (± se) of the site-occupancy probability was slightly higher than the observed one (0.433 ± 0.06). Contrary to observations, the model selection procedure suggested that this probability was similar across sectors (S1 = 0.54, S2 = 0.47, S3 = 0.49 and S4 = 0.55; Supplementary Fig. 1S(A)). Detection probabilities changed from 0.35 to 0.47, according to the sector considered (Supplementary Fig. 1S(B)). Model-averaged estimate (± se) was 0.492 (± 0.06). The model with the lowest AICc value was the one that assumed constant parameters (Supplementary Table 2S). This model was taken as a starting point to assess the influence of the covariates (see below; Table 3S).

**Figure 3:**
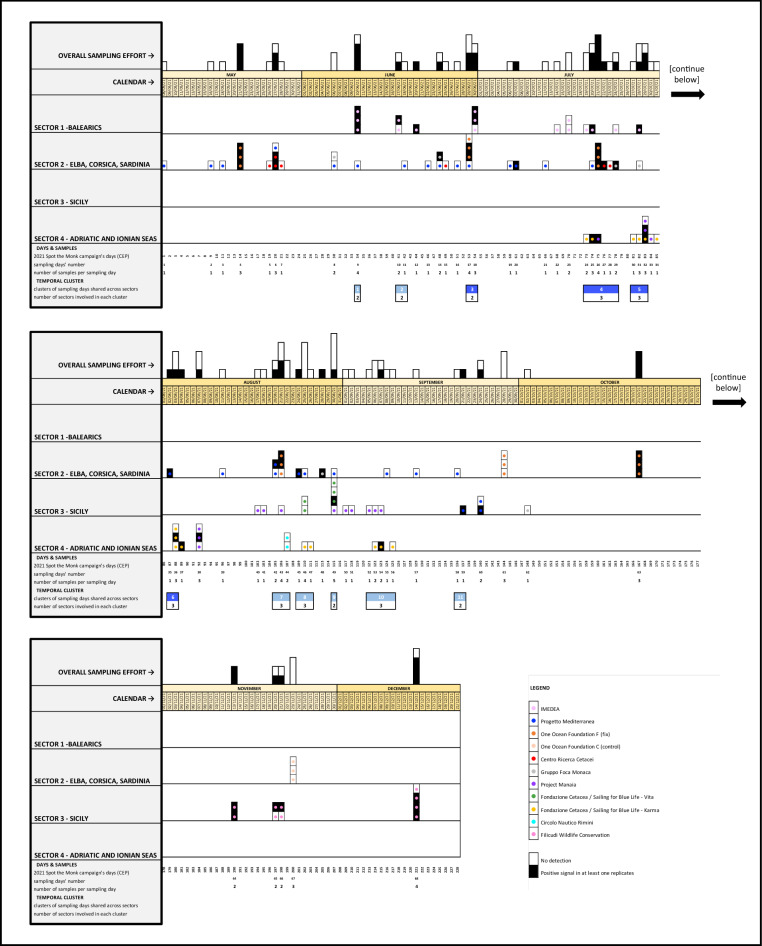
Calendar showing the chronological distribution of the samples (n = 126) collected during the Core Effort Period (CEP, from 08/05/21 to 14/12/21) both in the 4 sectors and overall (below and above the calendar bar respectively). The 8-months period has been split into 3 parts to enhance readability. Temporal-clusters, i.e. days or groups of days in which water samples were taken simultaneously in different sectors, are highlighted in the lower portion of each calendar row (blue boxes)—these were all concentrated between June and September, when the joint effort of several CS operators was highest. The number in the white part of each temporal-cluster box indicates the number of sectors simultaneously sampled. Temporal-clusters marked with darker blue (#3, #4, #5 and #6) are those in which samples were found positive in two or three sectors simultaneously.

**Figure 4:**
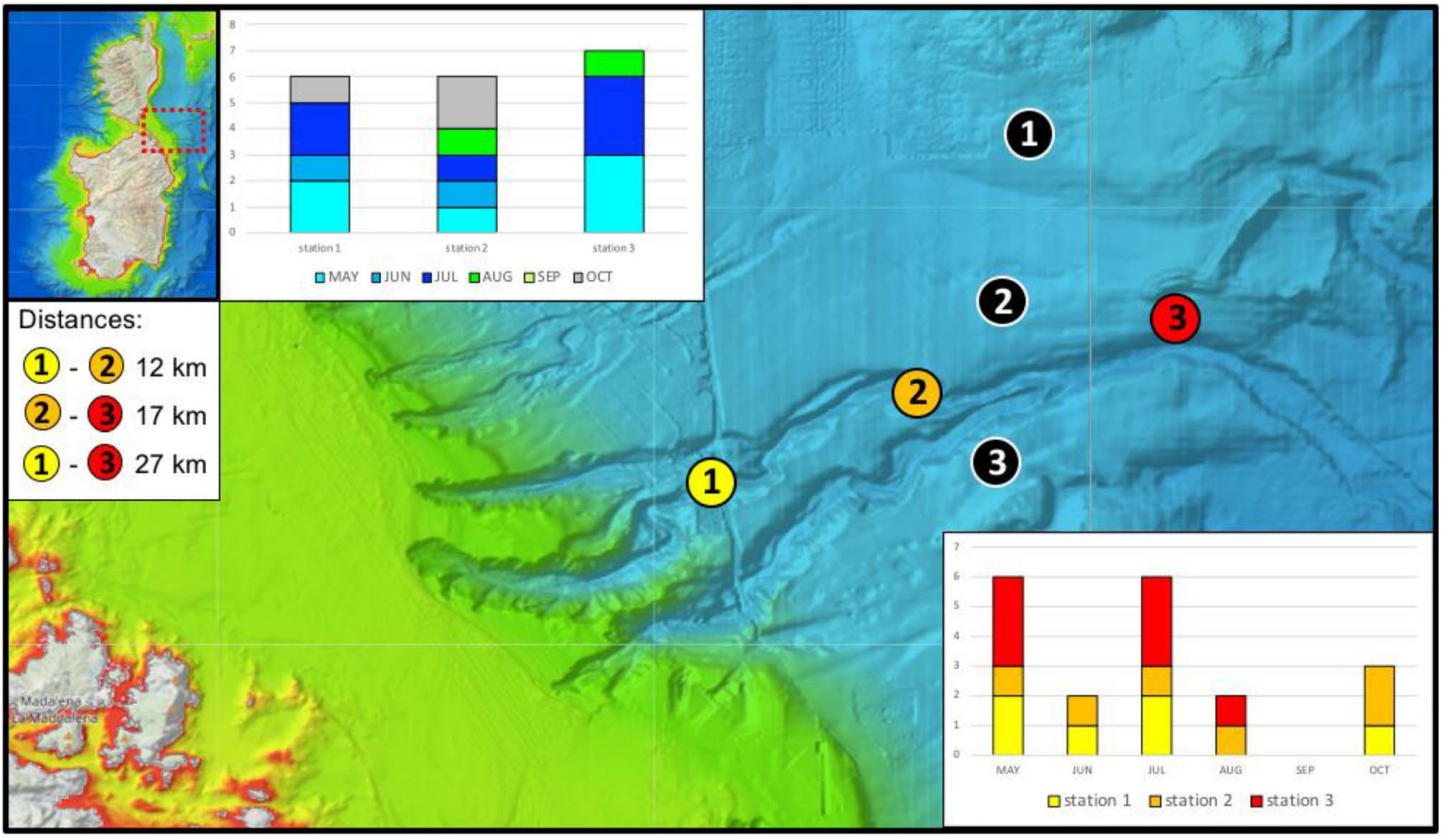
Focus-area sampling sites above the Caprera Canyon (area geographically located in the red dashed rectangle in the map in the left top corner). Coloured circles show the positioning of the three fixed sampling stations sampled monthly from May to October 2021. In black are the 3 control samples surveyed in Nov 2021. The number of positive replicates in the three study stations and across the six-month interval are plotted in the upper and lower graphs respectively. Maps retrieved from https://www.emodnet-bathymetry.eu/.

The analysis of spatio-temporal distances between pairs of observed positive samples within each of the six hotspots (Supplementary Fig. 2S), combined with the calendar of stacked data from all sectors (Fig. 3), allowed the identification of: (i) nested subsets of samples, collected in spatio-temporal proximity, and probably referable to the same individual/s from a single visit (e.g. the three positive samples collected along the island of Sa Dragonera the 10th of June 2021); (ii) areas habitually frequented by seals (i.e. regions in which positive signals were persistent over time), such as the Caprera Canyon (Fig. 4), the area of the Tuscan archipelago in the upper Tyrrhenian Sea, the Aeolian and Egadi archipelagos (Sicily) and the peninsula of Istria in the upper Adriatic; and (iii) the simultaneous presence of positive Monk Seal traces in various marine districts, either within the same or in different surveyed sectors (all these instances are detailed below in the Discussion, see Fig. 6).

### What about habitat quality, selection, use and seasonal occurrence?

Results from the replicated samples of the three fixed sampling stations in the Focus Area of Caprera Canyon suggested a regular use of this area by Monk Seals. Monk Seal DNA was detected in about two-thirds of the samples (66.67%; Fig. 4). Positive detections were found in at least two of the three fixed sampling stations each month, from May to October, with the exception of September (Fig. 4). In May and July positive signals were identified in all three monitored stations, with a more intense signal (number of positive replicates) in station 3, the furthest offshore one. Station 2, instead, was the one that gave the most regular positive results (in 5 out of 6 surveyed months), indicating a persistent presence of the Monk Seal in that area (Fig. 4).

**Figure 5:**
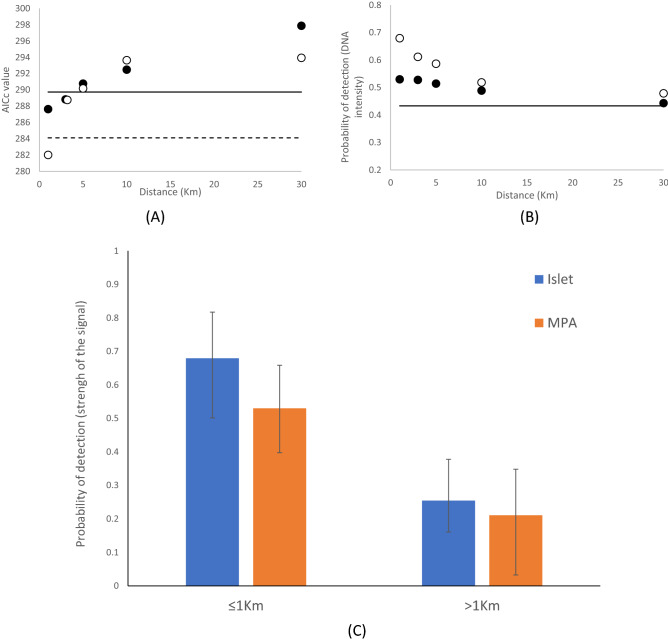
Relationship between incidence of positives and proximity to islets and MPAs. (**A**) AICc of models assuming a threshold value of the distance sets at 1, 3, 5, 10 and 30 km, respectively. Dots are the values for the variable ‘Islet’ (white symbol) and ‘MPA’ (solid symbol). The horizontal lines indicate a difference in AICc > 2 from the retained model of each variable. Threshold values for MPA was at 3 km, while for Islet was 1 km. (**B**) The probability of detection, equivalent to the strength of the eDNA signal, is a negative function of the distance from islets (white symbol) and marine protected areas (solid symbols). The horizontal line indicates the average value. (**C**) Detection probability (strength of the eDNA signal) increases near protected areas and islet.

The linear distances from islets and from protected areas did not significantly influence the occupancy probability, Ψ, (LRT: χ^2^_1_ = 0.663, p = 0.416 and χ^2^_1_ = 0.258, p = 0.612, respectively; Supplementary Table 3S). However, they did influence the detection probability, p. In particular the detection probability was greater near marine protected areas (χ^2^_1_ = 3.835, p = 0.05) but the model information theory suggested that this relationship was not linear. This is because, with the exception of model p(MPA_30) Ψ(.), all models assuming a threshold value of the distance from islets or MPA had a lower AICc values than the model assuming constant parameters, model p(.)Ψ(.) (Supplementary Table 3S and Fig. 5A,B). The threshold values of the two covariates, that is to say the distance beyond which detection is no longer significantly higher than the constant value, was different. The detection probability was significantly higher within 1 km from an islet and within 3 kms from marine protected areas (Supplementary Table 4S, Fig. 5C).

### Is citizen-science dataset valuable?

Most samples were collected by the general public and by non-profit organizations in an opportunistic way to gather precious information. However, a direct consequence was that processing times varied between samples. Water was filtered between 0 and 168 days after its collection (average: 24 days) and DNA was extracted on average 78 days after water collection (0 to 187 days). The processing time did not influence the occupancy probability. As expected, it negatively influenced the detection probability (i.e. signal intensity), but the relationship was not statistically significant (Supplementary Fig. 3S(C), SF: χ^2^_1_ = 0.46, p = 0.497; SE = χ^2^_1_ = 9.56, p = 0.097). The most delayed eDNA extraction occurred 187 days after water collection and returned a positive score.

## Discussion

Using the eDNA approach we detected monk seal DNA traces whose persistence in the marine matrix, although still not fully understood, is known to be limited over time. Collins et al.^33^, for example, suggested that the molecular trace duration at sea can last 2–3 days. A first clear result is that positive detections were found in each of the four geographic sectors, leading to the identification of six “hotspot” areas with high incidence or intensity of positive signals (Fig. 1 and Supplementary Fig. 4S). This was somehow unexpected considering the low number of sightings. Moreover, Monk Seal DNA has been found in over 50% of the sites sampled in highly touristic places, such as the Balearic Islands (Fig. 2B), an archipelago that receives ~ 12 million tourists per year. This is even more striking if we consider that the samples were collected at the beginning of the tourist season (June–July 2021) and that only 4 sightings have been reported since 2008, of which only one was validated by photographic records (F. Avellá *pers. comm.*). Also, even when correcting for detection probability, the proportion of sites with a positive outcome is an underestimation of Monk Seal presence if individuals visited the site a few weeks earlier. The incidence of positives, conditional to detection, in surveillance (S) samples (67.6%), collected in meticulously selected areas or habitats considered suitable for the Monk Seal, was more than double that found in opportunistic (O) samples (24.7%), collected during touristic cruises (therefore not dedicated to the seal study). It is interesting to note that in many surveyed sites (66%) the species had been reported to be present in historical times. This long-term site fidelity suggests a certain degree of heterogeneity across habitats and an active process of habitat selection by the seals. The distance to islets and marine protected areas influenced detection negatively, but not occupancy probability. The relationship between detection probability and distance to islets or marine protected area was not linear and detection was statistically significant only at short distances (< 1 km from islets and < 3 km from marine protected areas). In our case, detection probability is related to the intensity (quantity/quality) of the eDNA signal dissolved in the seawater contained in the bag. Although the duration of the signal depends on the organism from which it originates and the source and size of the particle^34^, it can be an indication that animals are visiting these sites intensively, or spend more time there. It should be noted that these two covariates were positively correlated because marine protected areas tend to include small archipelagos or off-shore islets. The high presence of eDNA near islets and restricted-access areas could suggest that these locations are beneficial for the protection of the Monk Seal, probably because they permit a constant regeneration of food supply and contribute to mitigate the wildlife-human conflicts. A stronger signal near islets or protected areas may also reflect the higher rate of dispersion of the eDNA molecules in open waters, where the sea currents are stronger compared to coastal ones. Only a large sampling effort in pelagic waters and a better understanding of marine mammal eDNA will shed light on this aspect.

**Figure 6:**
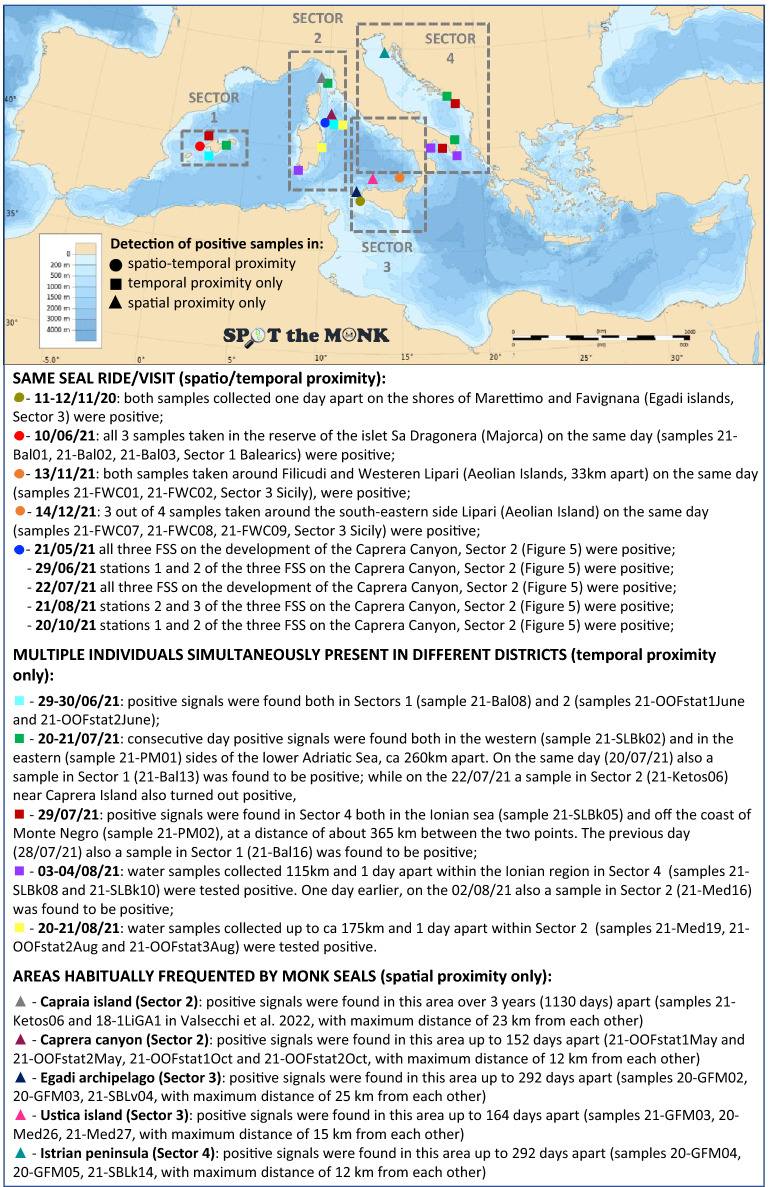
Results’ summary of the analysis of spatial/temporal distances between pairs of positive samples (refer to matrices in Supplementary Fig. 2S). Symbols of the same shape and colours indicate the position of samples collected in either spatial (triangle), temporal (square) or spatio-temporal (circle) proximity. The Mediterranean Sea Bathymetry map was retrieved from https://commons.wikimedia.org.

Despite the fact that targeted eDNA region cannot be used to identify individual animals, the distribution of spatio-temporal distances between pairs of positive samples (Supplementary Fig. 2S) allowed us to infer the simultaneous presence of multiple individuals or groups of individuals in distinct marine sectors and to detect areas that are habitually frequented by Monk Seals (Fig. 6). The identification of positive samples close in space and time provides additional clues on the reliability of the molecular technique, still novel to large-scale screenings in the wild. However, the spatio-temporal analysis of positive samples is also strongly dependent on the sampling effort and on the persistence of eDNA in sea water. The repeated samples in the Focus Area at Caprera Canyon showed a constant presence of Monk Seals in the waters overlooking the canyon (except in September). The persistence of positive Monk Seal traces in this area suggests that this marine district plays an important role for foraging, and this is consistent with findings for other marine mammals too^35^.

We present here a novel, non-invasive, citizen-science based approach to investigate the distribution and the habitat choice of an elusive and endangered pinniped. We also illustrate a possible analytical framework to obtained estimates of occupancy probability accounting for site-specific characteristics and detection probability. Despite the fact that some aspects related to the origin and fate of the biological traces still need to be understood, our work highlights the presence of the Monk Seal in all sectors of the central and western Mediterranean Sea. These results permit a re-visitation of the Monk Seal’s distribution map, showing how most of the sites that tested positive overlapped with its historical distribution. This long-term fidelity indicates the importance of these sites as resting or hunting grounds. We highlight six “hotspot areas” in the central and western Mediterranean Sea that deserve constant monitoring with repeated collections at regular intervals to better understand the use of the area/habitat by Monk Seals. We also showed that positive detections were clearly higher in the proximity of marine areas with restricted access, probably due to a high prey density, and to islets, that might provide resting stations. We detected two likely foraging corridors that deserve further research and protection: the Caprera Canyon and the Pianosa-Capraia continental edge. Finally, we showed that eDNA identification is a promising technique for large-scale, citizen-based projects. In particular, relying on citizens and non-profit organization increases the sample size and the spatial scale of the survey. It also contributes to a greater awareness of the general public on environmental issues and sustainable activities. The drawback is that the samples may be biased in terms of geographic areas (e.g. top tourist destinations may be more frequently sampled) and habitat type (samples are mostly, although not exclusively, collected along the coasts). It also produces many occasional samples that increase the extension and inconsistency of processing times. These negative aspects of citizen science can be overcome by relying on a protocol that maximizes the simultaneous collection of data and the spatial distribution of the sampling. It is interesting to note that the number of days occurring between water collection and water filtering or DNA extraction did not influence the occupancy nor the detection probability. This confirms that the DNA is a stable molecule and can help to design water collection and filtering in a more flexible way. For example, water can be collected in multiple sites at the same time (periodic bioblitz), but should not necessarily be filtered simultaneously or within a short time interval. However, further experiments are needed to measure the degradation of the DNA in sea water to fully understand and interpret the detection probability.

The current study provides a solid base for further and more specific research on Monk Seal habitat choice and distribution. This should rely on a sampling design that guarantees a long-term monitoring of sites and the simultaneous collection of sampling in multiple sites. It can also reach open sea areas with the collaboration of commercial and research vessels.

## Methods

### Study area and sample classification

We collected 135 samples in the central-western Mediterranean Sea, from the Adriatic Sea to the Balearic archipelago (Fig. 1). Samples were collected opportunistically except by research organizations that already operate in the area and those collected monthly from May to October 2021 at three fixed stations in a Focus Area (Caprera Canyon) in order to infer data on seasonal occurrence in this highly productive area. The majority of the samples (126 out of 135) were collected in 2021, from 08/05/21 to 14/12/21, hereafter defined as CEP (Core Effort Period). The nine remaining samples were collected from July 2020 to April 2021 (see supplementary Table 1S) either soon after a reported sighting or in habitats considered attractive to the Monk Seal (see below for sample classification). We divided the sampled area into 4 main sectors (Fig. 1), namely the Balearic archipelago (Sector 1), the islands of Corsica, Sardinia and Elba (Sector 2), Sicily (Sector 3) and the Adriatic and Ionian seas (Sector 4). The sector numbering follows the order in which the samples were analyzed, i.e. from west to east, therefore processing the samples with the lowest expected incidence of positives first, based on the current known distribution of the species. Moreover, the four sectors are sufficiently spaced out to be able to exclude the simultaneous presence of the same individuals in different sectors, facilitating an estimate, albeit approximate, of the incidence of the species within its distribution range.

The samples analyzed in this study were of four types (Supplementary Table 1S):

1. Opportunistic samples, randomly collected along the routes covered by partners (n = 73);
2. Focus-Area samples (n = 18) collected monthly at three fixed station sites (~ 30 kms apart) from the same area (Caprera canyon) from May to October 2021. Three additional control samples (n = 3) were collected on a transect perpendicular to the canyon.
3. Surveillance samples, collected at sites where seal sightings have been reported during the last decades or in habitats suitable to seals’ visit, such as rocky shores with superficial and submerged caves (n = 37; see Fig. 1).
4. Validation samples: marine water samples collected within 24–48 h from reported Monk Seal sightings (n = 4) in the Ionian and Tyrrhenian Seas.

### Marine water collection and processing

Seawater samples were collected and stored in Bags-in-Box containers, according to the BiBSS protocol^36^. All samples were collected from the most superficial layer of the sea (0–30 cm below sea level), either from a boat (n = 120) or from shore along the coastline (n = 14), with the exception of a single sample (20-GFM04) collected underwater at a depth of 6 m (Supplementary Table 1S). About 12 L of water were transferred into a light-resistant bag using sterilized devices, e.g. bilge pump or clean containers. In the majority of cases (77%), water filtering took place immediately (54%) or within 3–4 weeks after collection. During this time the bags containing samples were placed and stored in the shade—at a cool, but not freezing temperature. This ensured that cellular membranes of biological traces were maintained and not compromised by freezing, with consequent loss of the intracellular eDNA component. However, in about 23% of the cases, the storage time was longer than a month and in one exceptional case it was 168 days (average 23.6 days, se = 24.5). Each bag was divided into three 4-L aliquots, each filtered on a nitrocellulose filter with a porosity of 0.45 μm using the BioSart 100 filtration cylinders (Sartorius). The water sample was forced to pass through the filter thanks to the negative pressure created by means of a vacuum pump (Fisherbrand FB70155, Fisher Scientific) applied to the water-collection vacuum flask. Most samples (n = 109, 80.74%) produced 3 subsamples (filters A, B and C). In some cases, the subsamples were only two (A and B; n = 17, 12.59%) or one (A; n = 9, 6.67%), due to the partial loss of marine water during bag handling or filling. Filters were stored at -18ºC before the DNA extraction. The eDNA extraction took place from 3 to 151 days after filtering (average 52.3 days, se = 39.26).

Ethical approval is not relevant to this study, as all our samples consisted simply of marine water and no Monk Seal was even encounter during sample collection. Thus, all methods were carried out in accordance with relevant ethical guidelines and regulations.

### Molecular analysis

Here we outline the methodology used to extract the eDNA from the water samples, however for further details we refer readers to the description in Valsecchi et al^36^. The screening of the 135 samples analysed in this study involved the filtering of a total of 1431 L of sea water, filtered on a total of 370 membranes, the extracted DNA from which was run in triplicate for a total screening of 1100 (370 × 3) qPCR reactions (Supplementary Table 1S). Since the purpose of the study was to have an overview on the presence of the Monk Seal on a wide geographical area, we used a single locus, locus MarVer2, which was the best performing of the 3 loci described in Valsecchi et al.^32^. The locus choice was motivated for 3 reasons: (1) the high detectability rate; (2) the small size of the targeted amplicon (71 bp), particularly suitable for highly degraded DNA, such as eDNA; (3) the presence of a long string of unique bases (n = 8) at the 3' end of the Monk-Seal specific primer, significantly increasing its specificity. Quantitative Real Time PCR (qPCR) assays were performed on a Step One Plus (BIO-RAD) qPCR. The PCR temperature cycling conditions used were: 95 °C for 10 min; 40 cycles of denaturation at 95 °C for 15 s and annealing-elongation at 56 °C for 1 min; and a final dissociation stage. The amplification reaction contained 5.0 μl SsoFast EvaGreen Supermix with Low ROX (Bio-Rad), 0.1 μl of each [10 μM] primer solution, 2 μl DNA and 2.8 μl of Milli-Q water. Each of the eDNA sample replicates (1–3, according to the number of filters produced by each sample bag) were run in triplicate. For each run, one positive control sample, consisting of a 1:10^3^ diluted (216 ng/μl) and purified (Agencourt AMPure XP, Beckman Coulter) amplicon obtained from amplification of monk seal tissue, and no-template negative controls were also included in triplicate. The Ct (Cycle threshold) values were converted into counts. When the qPCR copy number output was below the LOQ but above the theoretical qPCR limit (three copies per reaction according to Bustin et al.^37^) but below the LOQ (according to Klymus et al.^38^) the qPCR copy number output was considered Detectable But Not Quantifiable (DBNQ). Species-specific amplification was confirmed by melting temperatures (TM) measured through the dissociation stage and equal to 71.5 ± 0.3 °C (see Valsecchi et al.^32^). There are currently no set criteria for the minimum number of eDNA qPCR technical replicates required to be amplified in order to confirm a positive result^39^. Therefore, consistently with other studies (e.g. Farrell et al.^40^), positive amplification of one or more technical replicates per sample was counted as positive detection. Amplification ratio (the proportion of positive amplification detection relative to attempted technical replicate reactions^40,41^ is reported for all study samples (Supplemental Table 1S). Thus, here replicates were used not as a certification of “genuine presence”, but rather a) in order to increase the chance of detecting rare molecules and b) for quantifying signal abundance. Similarly, since the purpose of the study was to find molecular evidence of the presence/absence of the Monk Seal, rather than the absolute quantification of its signal, which is irrelevant for the type of target organism (see Discussion), we reported both PQD (Positive Quantifiable Detections) and DBNQ outcomes as “positive signals”.

### Statistical analysis of molecular outcomes

To account for false negatives, i.e. the species is present but not detected, in site-occupancy studies, MacKenzie et al.^42^ proposed a likelihood-based framework to estimate the occupancy probability, Ψ, simultaneously with the detection probability, *p*. Because these parameters appear in the model likelihood, biological hypotheses on the factors influencing the parameters or the covariates influencing them can be directly tested through model information theory. The detection probability, *p*, is estimated through repeated surveys. In our case, the marine water sample collected in the bag (~ 12 L) has been filtered in three batches of about ~ 4 L each analysed independently. The environmental DNA (eDNA) was present in such a tiny amount that often only one or two filters from the same bag gave a positive score. Because a negative filter does not necessarily correspond to the absence of eDNA on a particular bag, each filter can be assumed as an independent survey of the site (bag). We coded eDNA score into site occupancy histories with three repeated surveys, one corresponding to each filter, and analysed the data using single-season occupancy model^42^ in program MARK v6.2^43^. To avoid pseudo-replication from repeated samples in FA, we retained only data from the mid period (July) and discarded the other replicates. Model selection procedure follow the AIC value^44^ corrected for sample size (AICc). The model with the lowest AICc value should be considered the best compromised between model deviance and model complexity. With small or sparse data several competing models might have similar AICc values (< 2 points). To account for model selection uncertainty, estimates from each sector were obtained by model averaging^43^. Finally, we wanted to investigate the influence of two sets of covariates, one set related to bag handling and DNA extraction and a second set related to site characteristics. In particular, for bag handling and DNA extraction we considered the time spent between water collection and water filtering (noted ‘SF’) and the time spent between water collection and eDNA extraction (noted ‘SE’). We assessed the effect of these two covariates only on the detection probability, *p*, because related to eDNA degradation. For site characteristics, we considered the distances from both the closest minor island (surface < 200 km^2^; note ‘Islet’ in model notation) and from the closest area with accessibility restriction (noted ‘MPA’ in model notation).

The latter included marine protected areas (mapped according to the site https://www.mapamed.org/) as well as military and industrial, e.g. oil/gas extraction platforms, areas localized using the Navionix application. To explore non-linear relationship we transformed these continuous covariates in a binary classification variable (1–0) considering a threshold distance, *θ*, with *θ* = 1, 3, 5, 10 and 30 km (a Monk Seal can travel ~ 40 km per day^45^. The binary variable took values of 0 for sites at a distance greater than *θ* and of 1 otherwise. Many sites close to marine protected areas were also located close to islets (correlation between MPA and Islet: p = 0.002). For this reason, these two variables were tested alone. The statistical significance level for each covariate was calculated using a Likelihood Ratio Test^43^.

Finally, we used observations conditional to detection to investigate the spatio-temporal distribution of the positive samples in order to detect multiple animals (or group of individuals) that are present simultaneously at site far apart or in different in sectors. We also identified, within our sampling calendar, days or groups of days in which water samples were taken simultaneously (i.e. same day plus ± 1) in different sectors.

## Supplementary Information

Supplementary Information.

## Data Availability

Details on sampling points, dates and distribution of positives are made available in the Supplementary Information file. Further datasets produced during the current study (e.g. DNA copy number outputs) are available from the corresponding author on request.
