# Author Correction: Playing “hide and seek” with the Mediterranean monk seal: a citizen science dataset reveals its distribution from molecular traces (eDNA)

**DOI:** 10.1038/s41598-023-33069-3

**Published:** 2023-04-12

**Authors:** Elena Valsecchi, Giacomo Tavecchia, Ginevra Boldrocchi, Emanuele Coppola, Denise Ramella, Livia Conte, Monica Blasi, Antonia Bruno, Paolo Galli

**Affiliations:** 1 Department of Environmental and Earth Sciences, University of Milano-Bicocca, Piazza della Scienza 1, 20126 Milan, Italy; 2 MaRHE Center, Magoodhoo Island, Faafu Atoll Republic of Maldives; 3 Mediterranean Institute for Advanced Studies (IMEDEA-CSIC/UIB), IMEDEA-C/Miquel Marquès, 21, 07190 Esporles, Balearics Islands Spain; 4 Department of Human Sciences, Innovation and Territory, University of Insubria, Via Valleggio 11, Como, Italy; 5 One Ocean Foundation, Via Gesù 10, Milan, Italy; 6 Gruppo Foca Monaca APS, Via Carlo Emery 47, 00188 Rome, Italy; 7 Filicudi Wildlife Conservation, Stimpagnato, 98055 Filicudi, Lipari (ME) Italy; 8 Department of Biotechnology and Biosciences, University of Milano-Bicocca, Piazza della Scienza 2, 20126 Milan, Italy

Correction to: *Scientific Reports* https://doi.org/10.1038/s41598-023-27835-6, published online 14 February 2023

The original version of this Article contained an error in Figure 1 where the light-brown triangle indicating Gruppo Foca Monaca (2020) and the yellow square indicating positive samples were omitted in the legend. The original Figure 1 and accompanying legend appear below.

**Figure 1:**
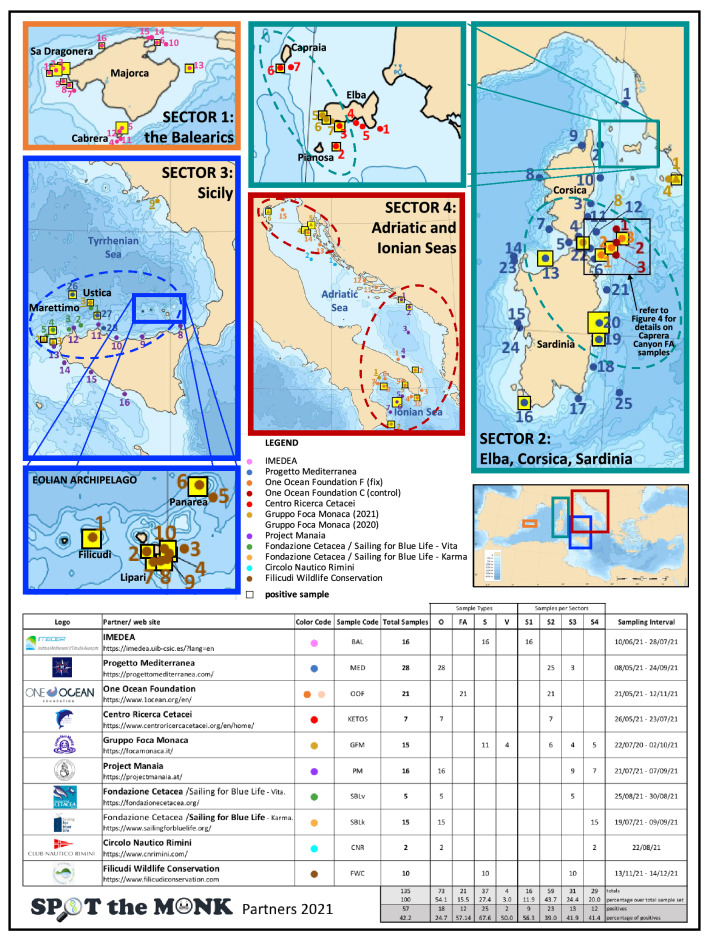
Maps of the four surveyed sectors showing the 120 points where samples (n = 135) were collected. Sectors’ frames are colour-coded and their geographical position in relation to the Mediterranean Sea is shown in the map at the bottom right. For Sectors 2 and 3 a sub-map (with the same colour-code as the sector) is given to illustrate the samples taken from restricted areas. Sampling points surrounded by a yellow square indicate samples that tested positive for the presence of Monk Seal DNA in at least one of the experimental replicates: the size of the yellow square is proportional to the number of positive responses (the bigger it is the stronger the signal). The dashed lines delimit areas (hotspots) where multiple positive samples were observed and which would deserve continuous monitoring. The four underscored sample-numbers indicate V samples (validation samples) collected after a reported sighting, under the GFM coordination. The bottom table lists all the Spot the Monk partners contributing to the collection of samples, and their sampling effort (detailed per sample category, sector and sampling period). The Mediterranean Sea Bathymetry map was retrieved, and subsequently cropped, from https://commons.wikimedia.org.

The original Article has been corrected.

