# Supplementary material for: Playing “hide and seek” with the Mediterranean monk seal: a citizen science dataset reveals its distribution from molecular traces (eDNA)
Source: Sci Rep. 2023 Feb 14;13:2610. doi: 10.1038/s41598-023-27835-6 (PMC9929094; doi:10.1038/s41598-023-27835-6)
Supplement: Supplementary file 1 — Supplementary Information. [file 41598_2023_27835_MOESM1_ESM.pptx]

## Slide 1
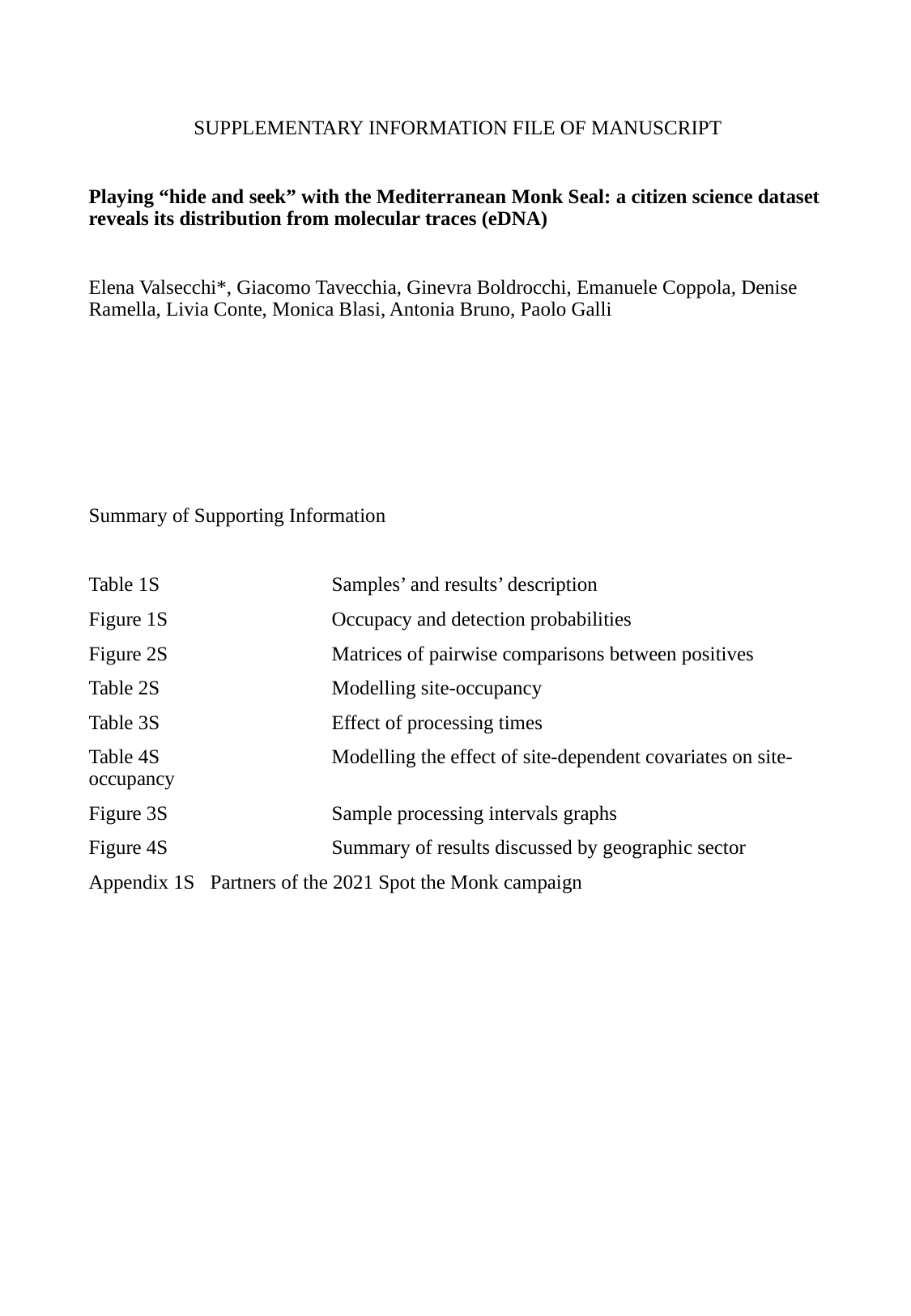

SUPPLEMENTARY INFORMATION FILE OF MANUSCRIPT
Playing “hide and seek” with the Mediterranean Monk Seal: a citizen science dataset reveals its distribution from molecular traces (eDNA)
Elena Valsecchi*, Giacomo Tavecchia, Ginevra Boldrocchi, Emanuele Coppola, Denise Ramella, Livia Conte, Monica Blasi, Antonia Bruno, Paolo Galli
Summary of Supporting Information
Table 1S		Samples’ and results’ description
Figure 1S		Occupacy and detection probabilities
Figure 2S		Matrices of pairwise comparisons between positives
Table 2S		Modelling site-occupancy
Table 3S		Effect of processing times
Table 4S		Modelling the effect of site-dependent covariates on site-occupancy
Figure 3S		Sample processing intervals graphs
Figure 4S		Summary of results discussed by geographic sector
Appendix 1S	Partners of the 2021 Spot the Monk campaign

## Slide 2
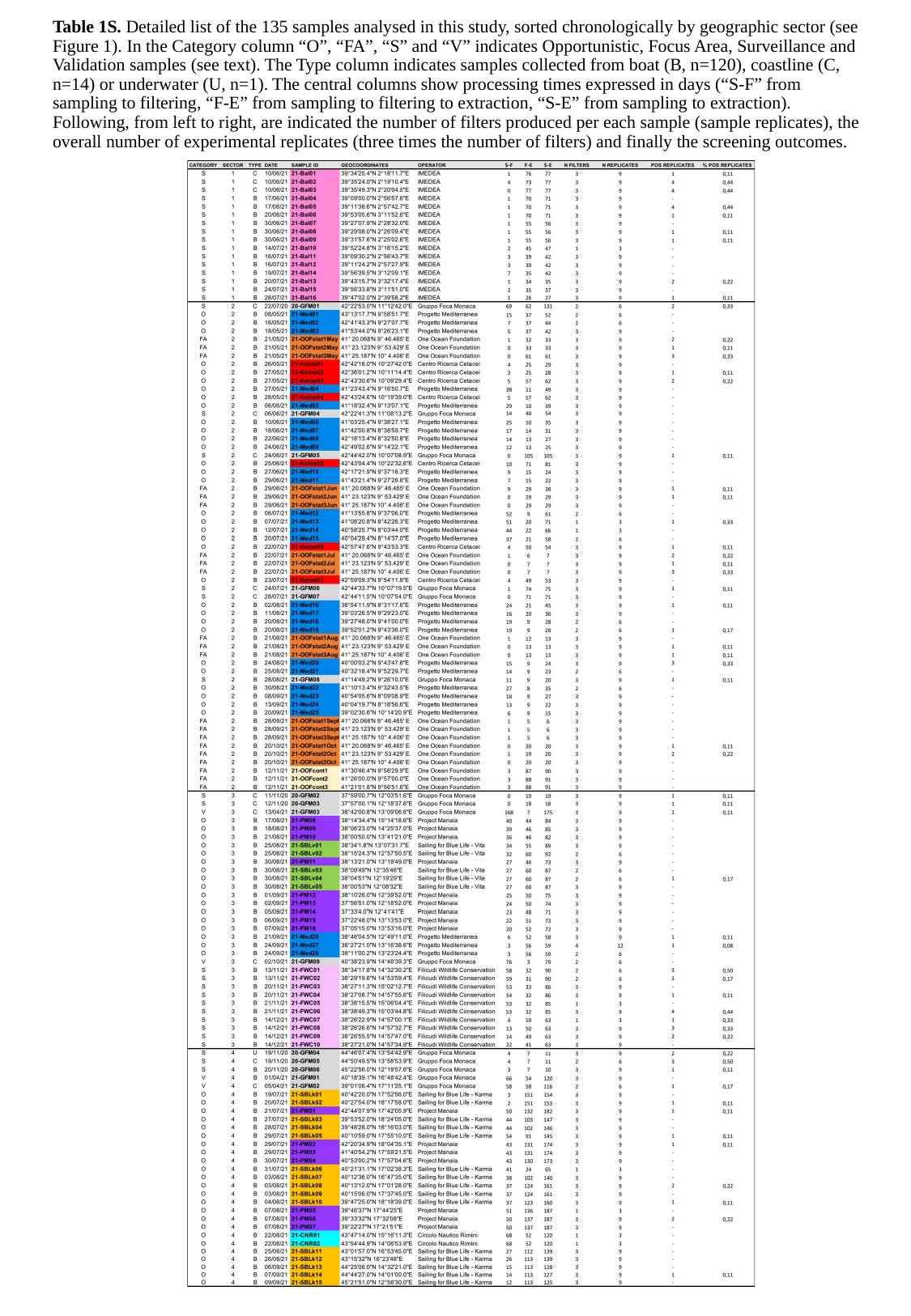

Table 1S. Detailed list of the 135 samples analysed in this study, sorted chronologically by geographic sector (see Figure 1). In the Category column “O”, “FA”, “S” and “V” indicates Opportunistic, Focus Area, Surveillance and Validation samples (see text). The Type column indicates samples collected from boat (B, n=120), coastline (C, n=14) or underwater (U, n=1). The central columns show processing times expressed in days (“S-F” from sampling to filtering, “F-E” from sampling to filtering to extraction, “S-E” from sampling to extraction). Following, from left to right, are indicated the number of filters produced per each sample (sample replicates), the overall number of experimental replicates (three times the number of filters) and finally the screening outcomes.

## Slide 3
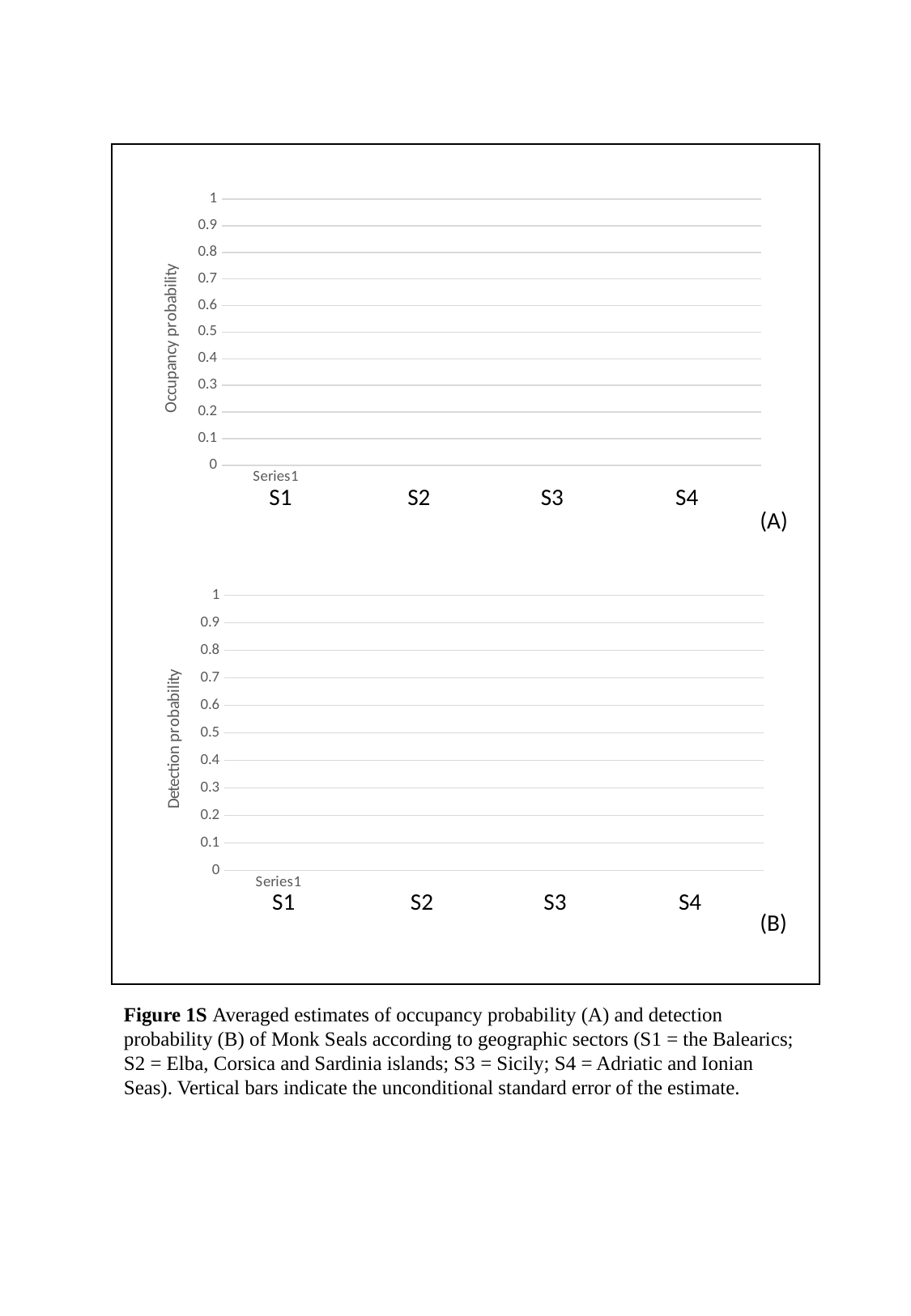

### Chart
| Category | PSI |
|---|---|
| | 0.5371113 |
| | 0.4713581 |
| | 0.4924455 |
| | 0.5548406 |S1 S2 S3	 S4
(A)
(B)
### Chart
| Category | p |
|---|---|
| | 0.4586966 |
| | 0.472351 |
| | 0.4392109 |
| | 0.3490707 |S1 S2 S3	 S4
Figure 1S Averaged estimates of occupancy probability (A) and detection probability (B) of Monk Seals according to geographic sectors (S1 = the Balearics; S2 = Elba, Corsica and Sardinia islands; S3 = Sicily; S4 = Adriatic and Ionian Seas). Vertical bars indicate the unconditional standard error of the estimate.

## Slide 4
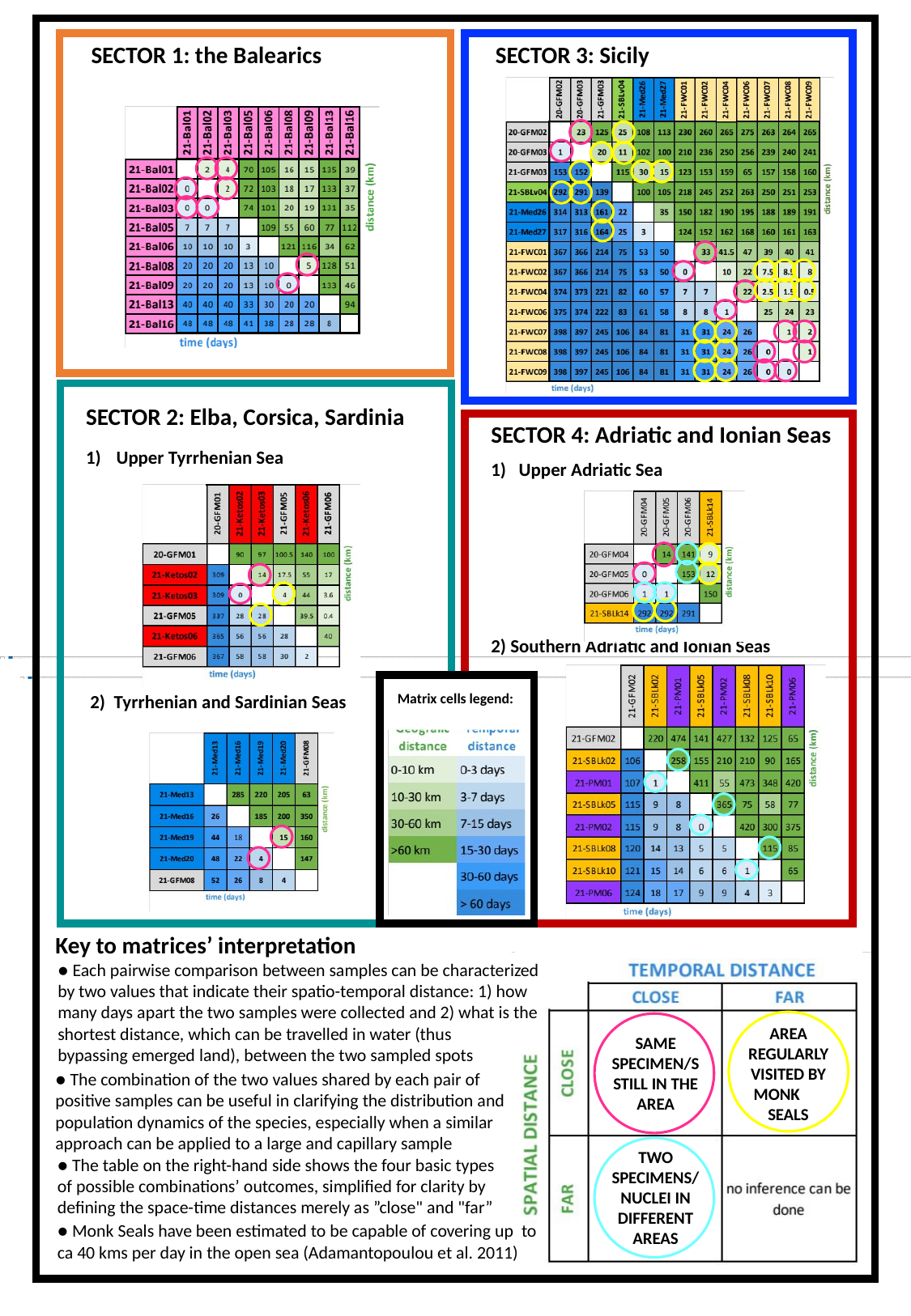

SECTOR 1: the Balearics
SECTOR 3: Sicily
SECTOR 2: Elba, Corsica, Sardinia
Upper Tyrrhenian Sea
 2) Tyrrhenian and Sardinian Seas
SECTOR 4: Adriatic and Ionian Seas
1) Upper Adriatic Sea
2) Southern Adriatic and Ionian Seas
Matrix cells legend:
Key to matrices’ interpretation
● Each pairwise comparison between samples can be characterized by two values that indicate their spatio-temporal distance: 1) how many days apart the two samples were collected and 2) what is the shortest distance, which can be travelled in water (thus
bypassing emerged land), between the two sampled spots
AREA REGULARLY VISITED BY MONK SEALS
SAME SPECIMEN/S STILL IN THE AREA
● The combination of the two values shared by each pair of positive samples can be useful in clarifying the distribution and population dynamics of the species, especially when a similar approach can be applied to a large and capillary sample
TWO SPECIMENS/ NUCLEI IN DIFFERENT AREAS
● The table on the right-hand side shows the four basic types of possible combinations’ outcomes, simplified for clarity by defining the space-time distances merely as ”close" and "far”
● Monk Seals have been estimated to be capable of covering up to ca 40 kms per day in the open sea (Adamantopoulou et al. 2011)

## Slide 5
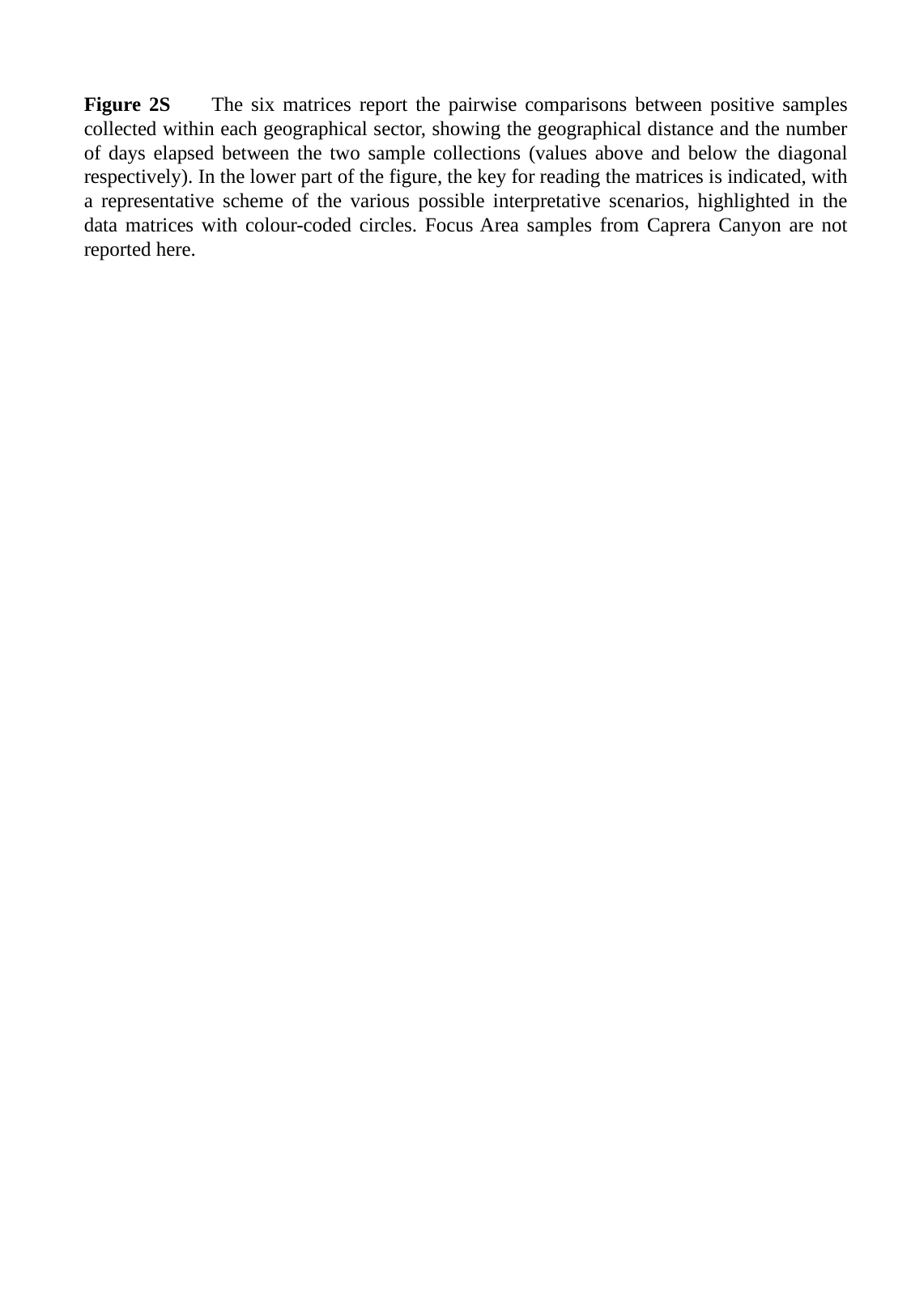

Figure 2S 	The six matrices report the pairwise comparisons between positive samples collected within each geographical sector, showing the geographical distance and the number of days elapsed between the two sample collections (values above and below the diagonal respectively). In the lower part of the figure, the key for reading the matrices is indicated, with a representative scheme of the various possible interpretative scenarios, highlighted in the data matrices with colour-coded circles. Focus Area samples from Caprera Canyon are not reported here.

## Slide 6
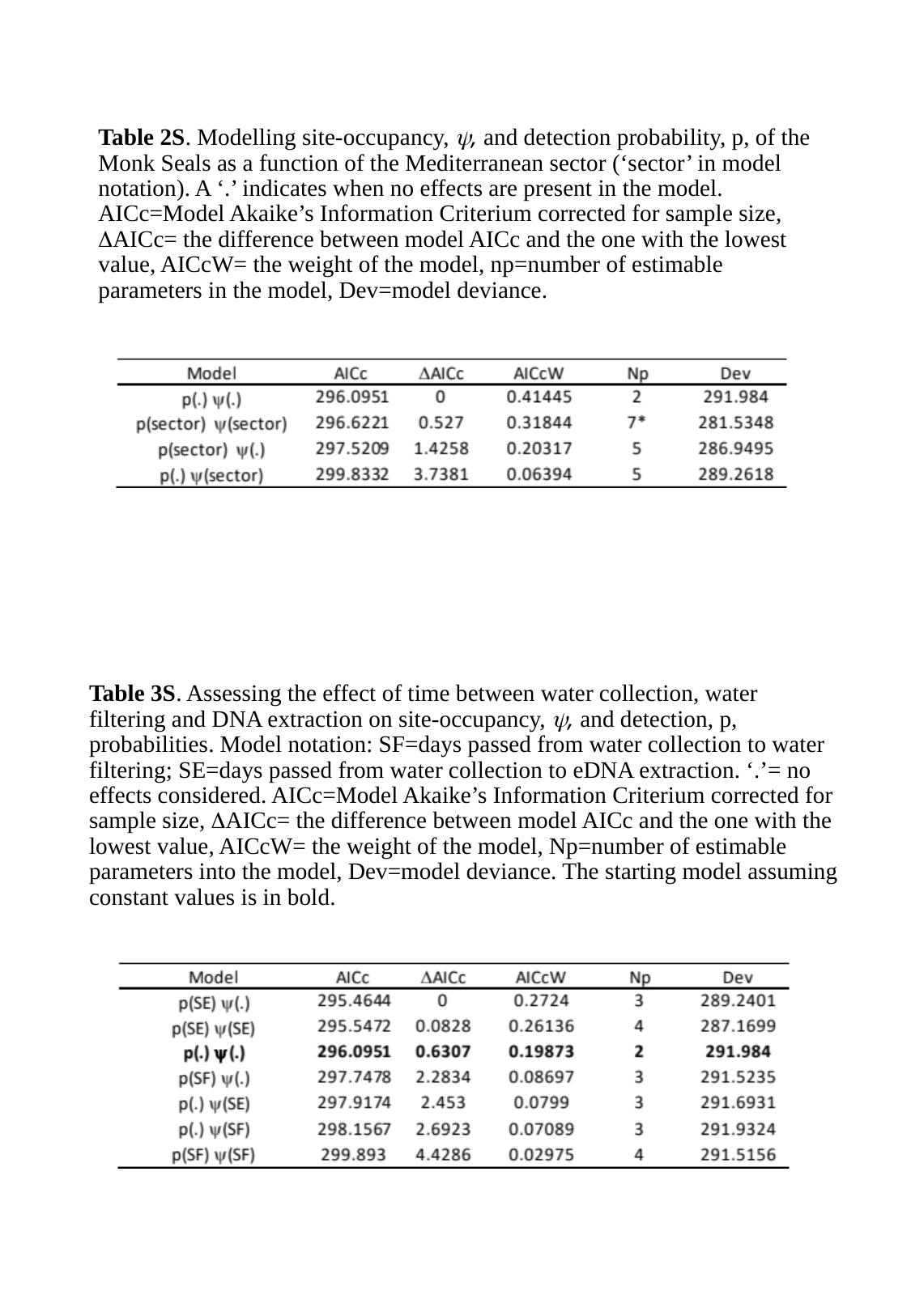

Table 2S. Modelling site-occupancy, y, and detection probability, p, of the Monk Seals as a function of the Mediterranean sector (‘sector’ in model notation). A ‘.’ indicates when no effects are present in the model. AICc=Model Akaike’s Information Criterium corrected for sample size, DAICc= the difference between model AICc and the one with the lowest value, AICcW= the weight of the model, np=number of estimable parameters in the model, Dev=model deviance.
Table 3S. Assessing the effect of time between water collection, water filtering and DNA extraction on site-occupancy, y, and detection, p, probabilities. Model notation: SF=days passed from water collection to water filtering; SE=days passed from water collection to eDNA extraction. ‘.’= no effects considered. AICc=Model Akaike’s Information Criterium corrected for sample size, DAICc= the difference between model AICc and the one with the lowest value, AICcW= the weight of the model, Np=number of estimable parameters into the model, Dev=model deviance. The starting model assuming constant values is in bold.

## Slide 7
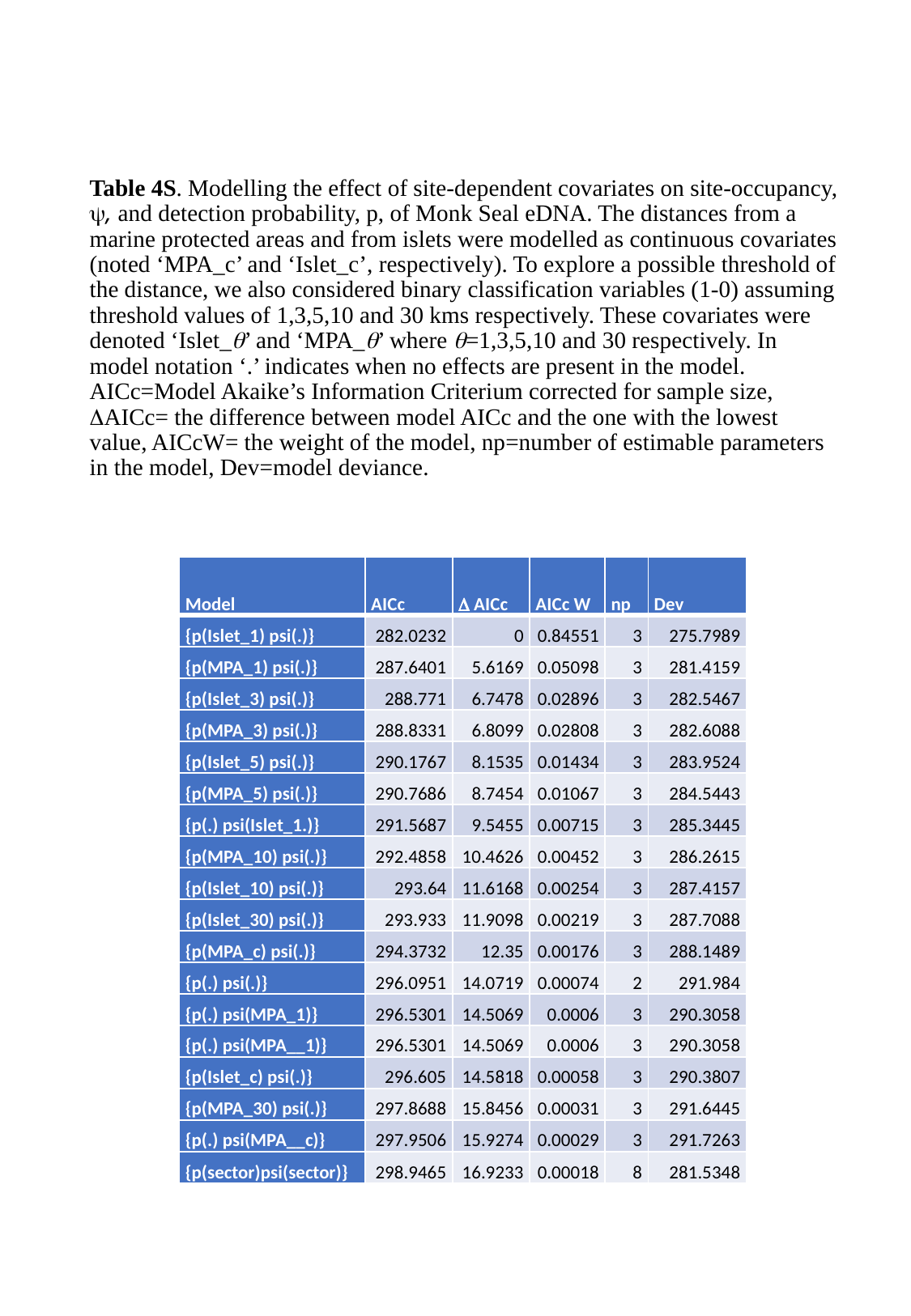

Table 4S. Modelling the effect of site-dependent covariates on site-occupancy, y, and detection probability, p, of Monk Seal eDNA. The distances from a marine protected areas and from islets were modelled as continuous covariates (noted ‘MPA_c’ and ‘Islet_c’, respectively). To explore a possible threshold of the distance, we also considered binary classification variables (1-0) assuming threshold values of 1,3,5,10 and 30 kms respectively. These covariates were denoted ‘Islet_q’ and ‘MPA_q’ where q=1,3,5,10 and 30 respectively. In model notation ‘.’ indicates when no effects are present in the model. AICc=Model Akaike’s Information Criterium corrected for sample size, DAICc= the difference between model AICc and the one with the lowest value, AICcW= the weight of the model, np=number of estimable parameters in the model, Dev=model deviance.
| Model | AICc | D AICc | AICc W | np | Dev |
| --- | --- | --- | --- | --- | --- |
| {p(Islet\_1) psi(.)} | 282.0232 | 0 | 0.84551 | 3 | 275.7989 |
| {p(MPA\_1) psi(.)} | 287.6401 | 5.6169 | 0.05098 | 3 | 281.4159 |
| {p(Islet\_3) psi(.)} | 288.771 | 6.7478 | 0.02896 | 3 | 282.5467 |
| {p(MPA\_3) psi(.)} | 288.8331 | 6.8099 | 0.02808 | 3 | 282.6088 |
| {p(Islet\_5) psi(.)} | 290.1767 | 8.1535 | 0.01434 | 3 | 283.9524 |
| {p(MPA\_5) psi(.)} | 290.7686 | 8.7454 | 0.01067 | 3 | 284.5443 |
| {p(.) psi(Islet\_1.)} | 291.5687 | 9.5455 | 0.00715 | 3 | 285.3445 |
| {p(MPA\_10) psi(.)} | 292.4858 | 10.4626 | 0.00452 | 3 | 286.2615 |
| {p(Islet\_10) psi(.)} | 293.64 | 11.6168 | 0.00254 | 3 | 287.4157 |
| {p(Islet\_30) psi(.)} | 293.933 | 11.9098 | 0.00219 | 3 | 287.7088 |
| {p(MPA\_c) psi(.)} | 294.3732 | 12.35 | 0.00176 | 3 | 288.1489 |
| {p(.) psi(.)} | 296.0951 | 14.0719 | 0.00074 | 2 | 291.984 |
| {p(.) psi(MPA\_1)} | 296.5301 | 14.5069 | 0.0006 | 3 | 290.3058 |
| {p(.) psi(MPA\_\_1)} | 296.5301 | 14.5069 | 0.0006 | 3 | 290.3058 |
| {p(Islet\_c) psi(.)} | 296.605 | 14.5818 | 0.00058 | 3 | 290.3807 |
| {p(MPA\_30) psi(.)} | 297.8688 | 15.8456 | 0.00031 | 3 | 291.6445 |
| {p(.) psi(MPA\_\_c)} | 297.9506 | 15.9274 | 0.00029 | 3 | 291.7263 |
| {p(sector)psi(sector)} | 298.9465 | 16.9233 | 0.00018 | 8 | 281.5348 |

## Slide 8
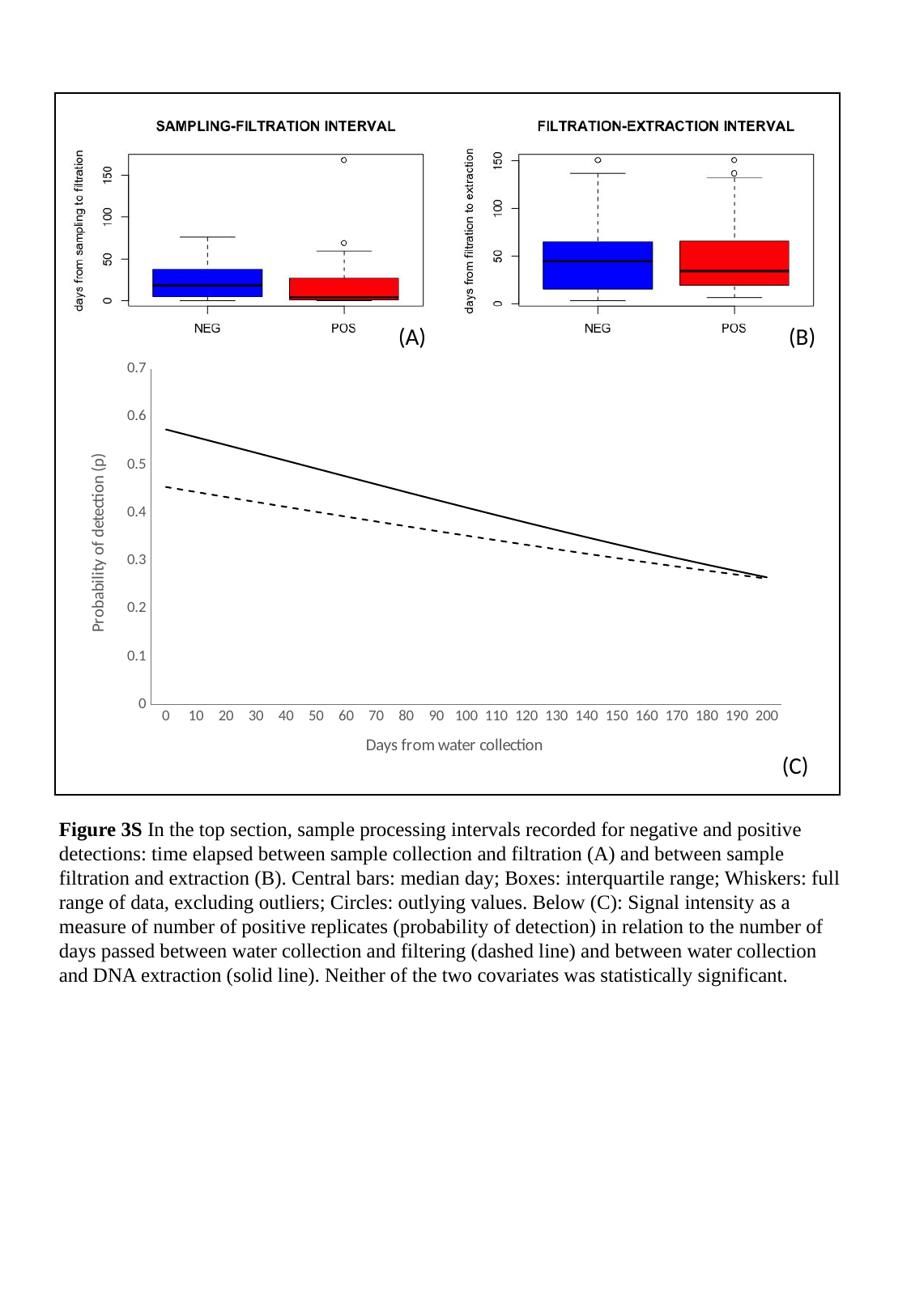

(A) (B)
### Chart
| Category | Sampling-to-Filtering | Sampling-to-Extraction |
|---|---|---|
| 0 | 0.45418769876648174 | 0.5743685909545913 |
| 10 | 0.4437052966630794 | 0.5581991540894067 |
| 20 | 0.4332727770374889 | 0.5419053666219558 |
| 30 | 0.42289907679617417 | 0.5255214509466405 |
| 40 | 0.41259292746320236 | 0.5090824008591258 |
| 50 | 0.40236282685848757 | 0.4926236851971385 |
| 60 | 0.39221701246974744 | 0.4761809428170115 |
| 70 | 0.38216343668363434 | 0.45978967445496677 |
| 80 | 0.37220974401916324 | 0.443484937119755 |
| 90 | 0.3623632504830401 | 0.4273010465839204 |
| 100 | 0.35263092514219124 | 0.41127129328842776 |
| 110 | 0.3430193739841221 | 0.39542767656163874 |
| 120 | 0.33353482611109336 | 0.3798006614980044 |
| 130 | 0.32418312228987084 | 0.3644189621698222 |
| 140 | 0.3149697058553437 | 0.34930935408691716 |
| 150 | 0.30589961594392767 | 0.3344965180067152 |
| 160 | 0.296977483011671 | 0.3200029163641358 |
| 170 | 0.2882075265725899 | 0.30584870276912923 |
| 180 | 0.27959355507518946 | 0.29205166423883416 |
| 190 | 0.2711389678195109 | 0.278627195116397 |
| 200 | 0.262846758803512 | 0.26558830099958847 |(C)
Figure 3S In the top section, sample processing intervals recorded for negative and positive detections: time elapsed between sample collection and filtration (A) and between sample filtration and extraction (B). Central bars: median day; Boxes: interquartile range; Whiskers: full range of data, excluding outliers; Circles: outlying values. Below (C): Signal intensity as a measure of number of positive replicates (probability of detection) in relation to the number of days passed between water collection and filtering (dashed line) and between water collection and DNA extraction (solid line). Neither of the two covariates was statistically significant.

## Slide 9
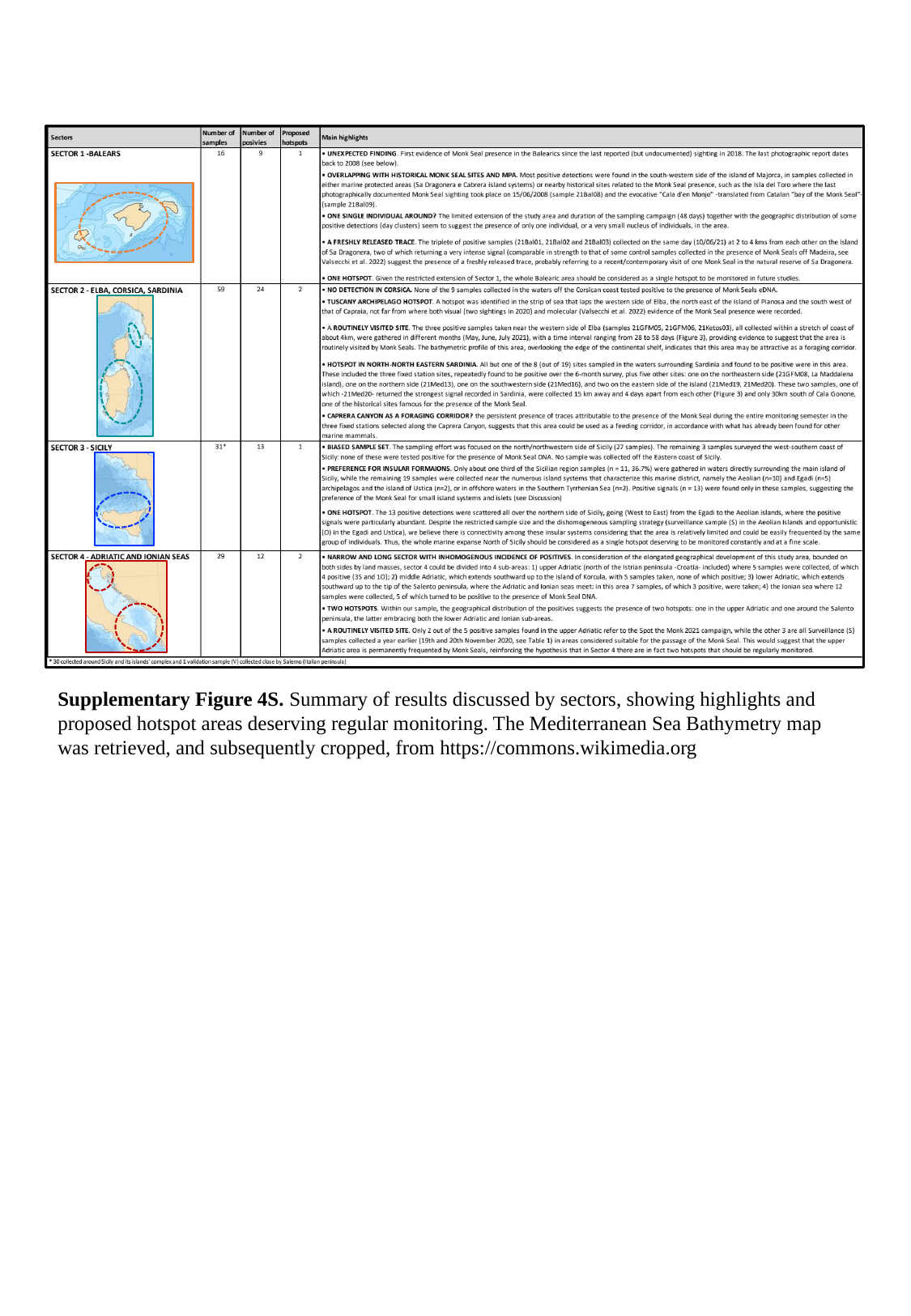

Supplementary Figure 4S. Summary of results discussed by sectors, showing highlights and proposed hotspot areas deserving regular monitoring. The Mediterranean Sea Bathymetry map was retrieved, and subsequently cropped, from https://commons.wikimedia.org

## Slide 10
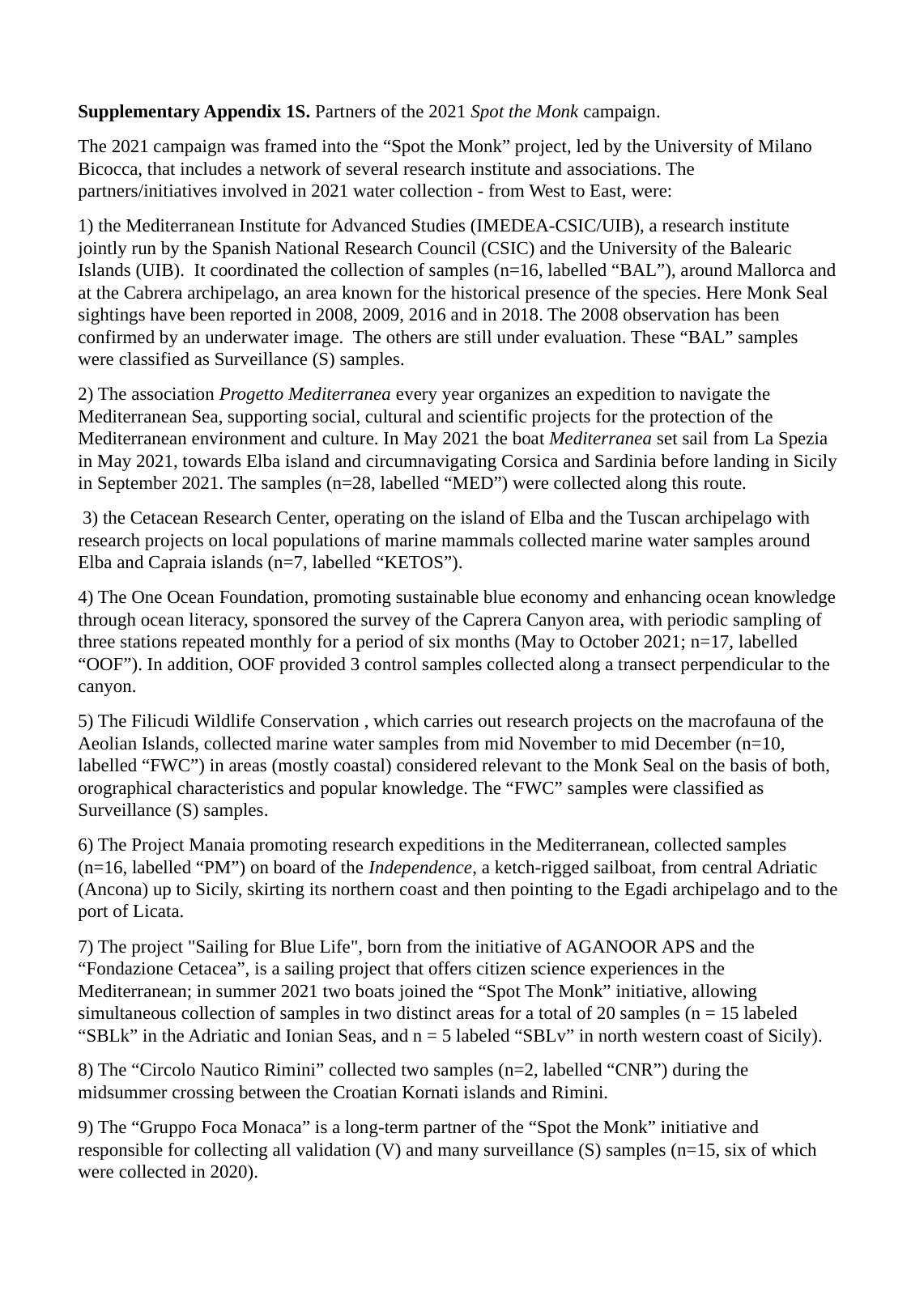

Supplementary Appendix 1S. Partners of the 2021 Spot the Monk campaign.
The 2021 campaign was framed into the “Spot the Monk” project, led by the University of Milano Bicocca, that includes a network of several research institute and associations. The partners/initiatives involved in 2021 water collection - from West to East, were:
1) the Mediterranean Institute for Advanced Studies (IMEDEA-CSIC/UIB), a research institute jointly run by the Spanish National Research Council (CSIC) and the University of the Balearic Islands (UIB). It coordinated the collection of samples (n=16, labelled “BAL”), around Mallorca and at the Cabrera archipelago, an area known for the historical presence of the species. Here Monk Seal sightings have been reported in 2008, 2009, 2016 and in 2018. The 2008 observation has been confirmed by an underwater image. The others are still under evaluation. These “BAL” samples were classified as Surveillance (S) samples.
2) The association Progetto Mediterranea every year organizes an expedition to navigate the Mediterranean Sea, supporting social, cultural and scientific projects for the protection of the Mediterranean environment and culture. In May 2021 the boat Mediterranea set sail from La Spezia in May 2021, towards Elba island and circumnavigating Corsica and Sardinia before landing in Sicily in September 2021. The samples (n=28, labelled “MED”) were collected along this route.
 3) the Cetacean Research Center, operating on the island of Elba and the Tuscan archipelago with research projects on local populations of marine mammals collected marine water samples around Elba and Capraia islands (n=7, labelled “KETOS”).
4) The One Ocean Foundation, promoting sustainable blue economy and enhancing ocean knowledge through ocean literacy, sponsored the survey of the Caprera Canyon area, with periodic sampling of three stations repeated monthly for a period of six months (May to October 2021; n=17, labelled “OOF”). In addition, OOF provided 3 control samples collected along a transect perpendicular to the canyon.
5) The Filicudi Wildlife Conservation , which carries out research projects on the macrofauna of the Aeolian Islands, collected marine water samples from mid November to mid December (n=10, labelled “FWC”) in areas (mostly coastal) considered relevant to the Monk Seal on the basis of both, orographical characteristics and popular knowledge. The “FWC” samples were classified as Surveillance (S) samples.
6) The Project Manaia promoting research expeditions in the Mediterranean, collected samples (n=16, labelled “PM”) on board of the Independence, a ketch-rigged sailboat, from central Adriatic (Ancona) up to Sicily, skirting its northern coast and then pointing to the Egadi archipelago and to the port of Licata.
7) The project "Sailing for Blue Life", born from the initiative of AGANOOR APS and the “Fondazione Cetacea”, is a sailing project that offers citizen science experiences in the Mediterranean; in summer 2021 two boats joined the “Spot The Monk” initiative, allowing simultaneous collection of samples in two distinct areas for a total of 20 samples (n = 15 labeled “SBLk” in the Adriatic and Ionian Seas, and n = 5 labeled “SBLv” in north western coast of Sicily).
8) The “Circolo Nautico Rimini” collected two samples (n=2, labelled “CNR”) during the midsummer crossing between the Croatian Kornati islands and Rimini.
9) The “Gruppo Foca Monaca” is a long-term partner of the “Spot the Monk” initiative and responsible for collecting all validation (V) and many surveillance (S) samples (n=15, six of which were collected in 2020).
